# Circadian analysis of myocardial infarction incidence in an Argentine and Uruguayan population

**DOI:** 10.1186/1471-2261-6-1

**Published:** 2006-01-09

**Authors:** Carlos E D'Negri, Leonardo Nicola-Siri, Daniel E Vigo, Luis A Girotti, Daniel P Cardinali

**Affiliations:** 1 Consejo Nacional de Investigaciones Científicas y Técnicas, Argentina; 2 División de Cardiología, Hospital Ramos Mejía, Buenos Aires, Argentina; 3 Laboratorio de Bioelectricidad, Escuela de Ingeniería – Bioingeniería, Universidad Nacional de Entre Ríos, Argentina; 4 Departamento de Fisiología, Facultad de Medicina, Universidad de Buenos Aires, Argentina

## Abstract

**Background:**

The occurrence of variations in the spectrum of cardiovascular disease between different regions of the world and ethnic groups have been the subject of great interest. This study report the 24-h variation of myocardial infarction (MI) occurrence in patients recruited from CCU located in Argentina and Uruguay.

**Methods:**

A cohort of 1063 patients admitted to the CCU within 24 h of the onset of symptoms of an acute MI was examined. MI incidence along the day was computed in 1 h-intervals.

**Results:**

A minimal MI incidence between 03:00 and 07:00 h and the occurrence of a first maximum between 08:00 and 12:00 h and a second maximum between 15:00 and 22:00 h were verified. The best fit curve was a 24 h cosinor (acrophase ~ 19:00 h, accounting for 63 % of variance) together with a symmetrical gaussian bell (maximum at ~ 10:00 h, accounting for 37 % of variance). A similar picture was observed for MI frequencies among different excluding subgroups (older or younger than 70 years; with or without previous symptoms; diabetics or non diabetics; Q wave- or non-Q wave-type MI; anterior or inferior MI location). Proportion between cosinor and gaussian probabilities was maintained among most subgroups except for older patients who had more MI at the afternoon and patients with previous symptoms who were equally distributed among the morning and afternoon maxima.

**Conclusion:**

The results support the existence of two maxima (at morning and afternoon hours) in MI incidence in the Argentine and Uruguayan population.

## Background

More than 50 years ago, Pell and D'Alonzo first demonstrated the existence of a peak in the morning hours in a study of acute myocardial infarction (MI) in a large industrial population [1]. Subsequent population studies showed a non-uniform circadian distribution of events with a morning peak [2-11] and sometimes a smaller afternoon peak [3,4,12]. A double-peak distribution of MI was reported more than a decade ago [13,14] with fatal infarctions being prevalent in the morning span [15]. Moreover, a temporal pattern characterized by a same double-peak curve has been found for both ischemic and hemorrhagic stroke [16-18], rupture/dissection of aortic aneurysms [19] and peripheral artery embolization [20].

The occurrence of variations in the spectrum of cardiovascular disease between different regions of the world and ethnic groups have been the subject of great interest [21-25]. There are also considerable variations in the prevalence of cardiovascular risk factors (smoking, hypertension, hyperlipidemia and diabetes) among different ethnic groups [26]. In a recent paper examining whether ethnicity influences the circadian pattern of acute MI, a significantly higher number of acute MI onsets occurring between midnight and noon was observed in British Caucasians and Indo-Asians, whereas in Mediterranean Caucasians most of the acute MI events happened between noon and midnight [25].

This prompted us to examine the MI incidence along the day in the data obtained from GEMICA Study database, a prospective, multicenter, randomized, placebo-controlled, double blind, trial design to assess the effect of amiodarone on mortality after MI in an Argentine and Uruguayan population. The results of the GEMICA study were published elsewhere [27].

## Methods

### Population examined

GEMICA was launched in March 1994 and completed in July 1995 [27]. Patients were screened and recruited from 63 CCU in Argentina, and from 2 in Uruguay. The protocol was reviewed and approved by an independent external Safety and Monitoring Board of the Study as well as by the local Institutional Ethics Committee of each participating center.

Every patient who was hospitalized within the first 24 h after the onset of MI was screened. Diagnosis of infarction was based on the confirmation of two out of the three accepted criteria: i) prolonged (longer than 30 min) typical chest pain not responding to nitroglycerin, ii) sustained ST segment changes and/or appearance of new Q waves; iii) doubling of the blood creatine-phosphokinase normal value. Table 1 summarizes comorbidities and concomitant therapy in the sample of patients examined. Criteria for eligibility of patients were detailed elsewhere [27].

**Table 1:** Patient comorbidities and concomitant therapy

**Comorbidity**	
Hypertension	541 (53.7%)
Diabetes	174 (16.4%)
Tobacco	475 (44.7%)
Previous Myocardial Infarction	158 (14.9%)

**Concomitant Therapy**	

Beta blocker	679 (63.9%)
ACE inhibitor	372 (35%)
Amiodarone	542 (51%)
Thrombolytic	652 (61.3%)
PTCA	130 (12.2%)
CABG	90 (8.5%)

Shown are the number of patients and percentage. For further information on the population studied see [27]

### Timing of MI onset

The time each patient had suffered his/her MI was recorded up to within ± 0.5 h by interrogation of the patients or of their close relatives. MI incidence along the day was analyzed on total population and after dividing the population in several mutually excluding subgroups, as follows: (i) older than 70 years, 70 or less years; (ii) with or without previous symptoms; (iii) diabetics or non diabetics; (iv) Q wave- or non-Q wave-type MI; (v) anterior or inferior MI location.

To quantify the time dependence of MI incidence, the 24 h period was divided into 1 h-intervals. Observed cases of MI onset in each time interval were expressed as percent incidence per hour (%/h) after normalizing the data by dividing the observed cases by N (the number of patients in total population or in each subgroup).

### Statistical analysis

Periodicity in occurrence of MI episodes along the day was assessed by comparing the distribution of MI incidence against a theoretical uniform distribution. The total population, as well as every subgroup, was separately analyzed. Differences in periodicity between each pair of mutually excluding subgroups were analyzed by using Chi-square test on 2 × 24 contingency tables.

Least squares regression analysis on MI incidence was performed for the total population and for every subgroup, using the following fitting function for the probability density **y(t) (%/h)**:

**y(t) **= A + B * cos(ω_24 _* (t -t_acrophase_)) + (C/((2*Π)^1/2 ^* σ)) * exp-((t-t_m_)^2^/(2*σ^2^))     [1]

The first component, A + B * cos(ω_24 _* (t -t_acrophase_)) is a cosinor function, i.e. a constant A (%/h) plus a harmonic oscillation around A with amplitude B (%/h), period 2 * Π/ω_24 _= 24 h and maximum at the acrophase t_acrophase _(hh:mm). By integrating over 24 h, the probability of cosinor component (%) resulted in A (%/h) times 24 h. The second component in [1] is a normal probability density function (gaussian) with t_m _(hh:mm) as mean value and σ (hh:mm) as SD. Gauss distribution was scaled to obtain C (%) for gaussian component.

Being y(t) a sum of probability densities, a fitting procedure was imposed to reject any negative values for every component and to comply with the normalization constraint A * 24 h + C = 100%. Fitted constants were expressed as mean ± SEM. Goodness of fit was checked through the correlation coefficient **r**. A minimum value of 0.7 (70%) was set to accept a fit. The temporal constants t_acrophase_, t_m_, and σ were compared between mutually excluding subgroups, and also for every subgroup against total population. Differences between means (m_i _- m_k_) were analyzed under the assumption of means being independent and normally distributed. On each case, the adimentional variable z = (m_i _- m_k_)/(se_i_^2 ^+ se_k_^2^)^1/2 ^was calculated, and the two-tailed p(z) value was obtained from a normal probability table.

Total probabilities for cosinor and gaussian components were not independent values. In consequence, differences between A * 24 h and C were analyzed for every subgroup, and also in the total population, by comparison against the half probability value 0.5 (i.e., 50%). A test of differences between two means modified for one-tailed p(z), was used. Differences were considered non-significant if p ≥ 0.05.

## Results

**Figure 1:**
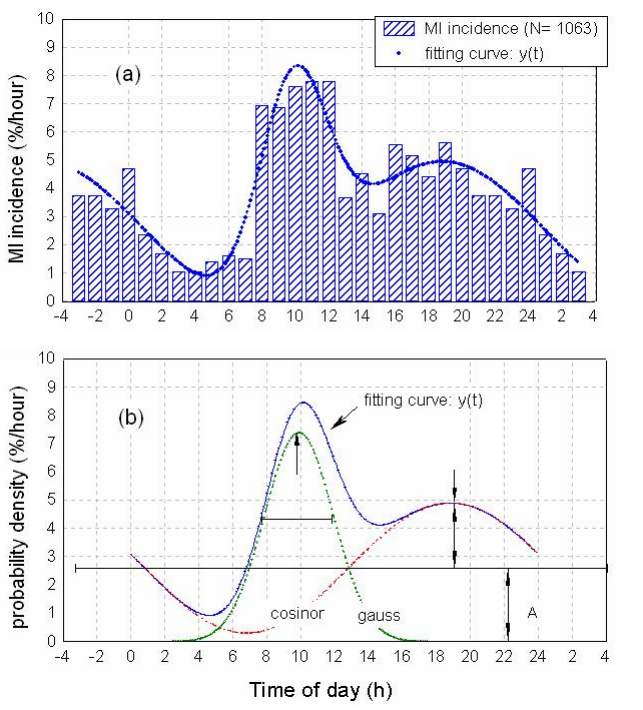
Circadian periodicity in MI incidence in the population examined. Upper panel (a): Bars indicate MI incidence (%/h) values, calculated as (100 * MI's per hour/total N). Curve fitting = cosinor + Gaussian for n = 24 time points was calculated as described in Methods. Values for 21:00 – 24:00 h (left) and 01:00 – 03:00 h (right) are duplicated to illustrate fitting function. Lower panel (b): Probability density functions (%/h) for MI incidence in total population. Cosinor and gaussian components are shown separately to illustrate the contribution of each parameter in fitting the function y(t).

The group of 1063 patients was analyzed as a total population and after division in excluding subgroups as described in Methods. Figure 1-a depicts the fitted curve **y(t) **superimposed to the observed hourly MI percentage. Inspection of data indicates minimal MI incidence between 03:00 and 07:00 h and the occurrence of a first peak between 08:00 and 12:00 h and a second peak between 15:00 and 22:00 h. This second peak occurred about 12 h after the minimal MI incidence; thus, a 24 h cosinor fitting was consistent with this observation. In addition, the morning maximum (between 08:00 and 12:00 h) resembles a symmetrical bell located over the rising flank of the cosinor curve; therefore, a gaussian component was added to the fitting function.

The bimodal distribution adopted relied on the existence of significant differences between morning and evening maxima and the minima observed before sunrise and at early afternoon. For the intervals 02:00 – 07:00 h and 08:00 – 12:00 h, each hourly MI incidence differed by more than 1 SD from daily mean (4 ± 2) %/h. In the case of the 13:00 – 24:00 h interval, mean hourly MI incidence was 4.4 ± 0.9 %/h, with values at 15:00, 16:00 and 19:00 h differing by >1 SD from the mean. This supported the existence of a minimum at 13:00 – 15:00 h and a maximum at 16:00 – 20:00 h, significantly above random noise

Figure 1-b shows the cosinor and gaussian components of the same population, as well as **y(t)**, to illustrate the contribution of each parameter in fitting the function. Overall, the fitted function accounted for 84.6 % of variance. Amplitude values for the cosinor component were A = (2.6 ± 0.3) %/h and B = (2.3 ± 0.4) %/h with an acrophase (t_acrophase_) of 18.8 ± 0.6 h (i.e., 18:49 ± 0:25 h). Integration over 24 h yielded a probability of 63 ± 7 % for the cosinor component (Fig. 1-b). The gaussian component exhibited a maximum (t_m_) at 9.9 ± 0.2 h (i.e., 09:52 ± 0:14 h) with a standard deviation (σ) of 2.0 ± 0.3 h. The probability of the gaussian component was 37 ± 7 %.

**Figure 2:**
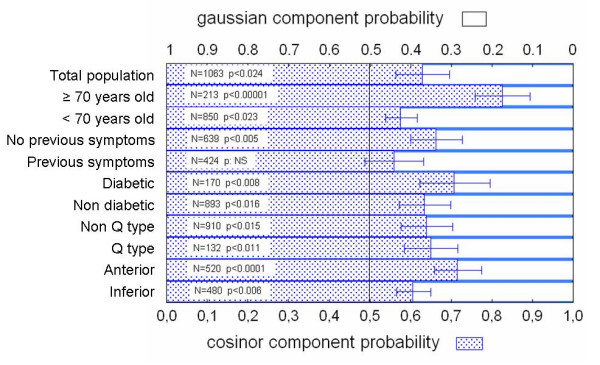
Probability of cosinor and gaussian components for total population and the excluding subgroups. Shown are the means ± SEM. Statistical comparisons of means against 0.5 (50%, see Methods for explanation) are shown. NS: non significant.

**Figure 3:**
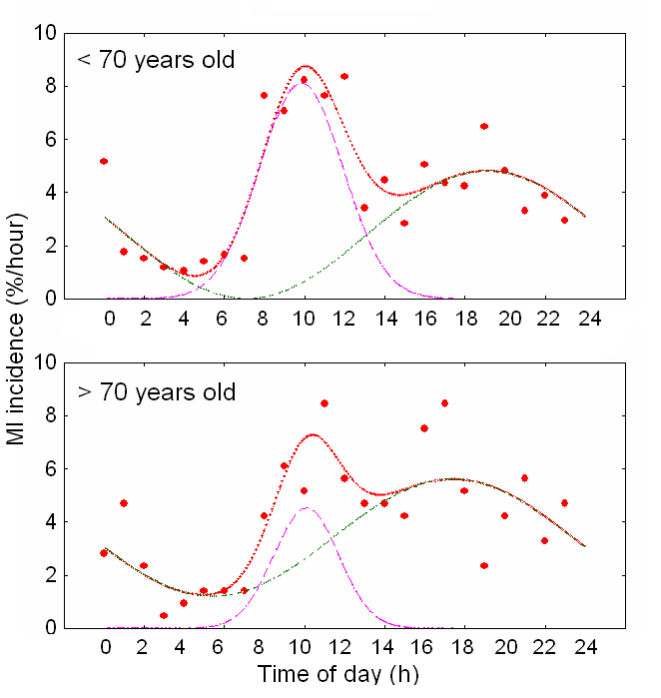
Circadian periodicity in MI incidence in patients younger and older than 70 years old. Probability density functions (%/h) for MI incidence are shown. Cosinor and gaussian components are depicted separately to illustrate the contribution of each parameter in fitting the function y(t).

**Figure 4:**
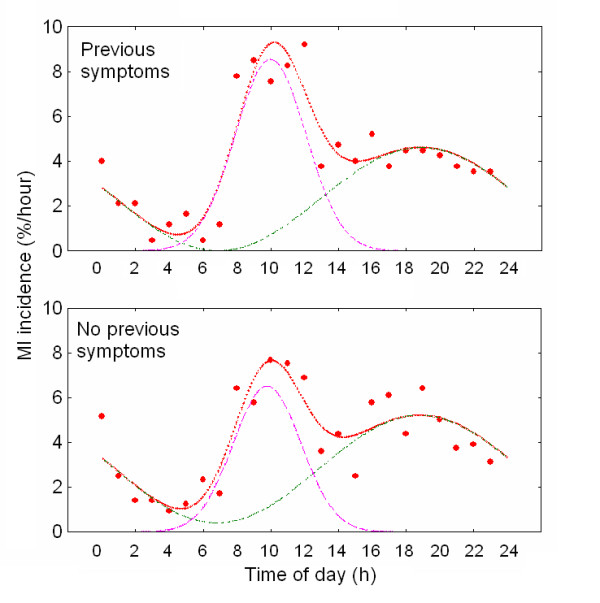
Circadian periodicity in MI incidence in patients with or without previous symptomatology. Probability density functions (%/h) for MI incidence are shown. Cosinor and gaussian components are depicted separately to illustrate the contribution of each parameter in fitting the function y(t).

When the analysis depicted in Fig. 1 was applied to MI incidence for the several excluding subgroups examined, a similar picture to the total population was obtained. Maxima in MI incidence were found at the morning (08:00 – 13:00 h) and at evening (~18:00 h) with a nadir at 02:00 – 07:00 h and secondary minima at 13:00 – 15:00 h. For every studied subgroup a 24 h cosinor plus a gaussian function described adequately the observed values. This is summarized in Additional file 1 and Fig. 2, in which MI probability values for the total population and for the excluding subgroups are shown. The correlation coefficients ranged from 0.79 to 0.92 and curve fitting explained 62 – 85 % of the observed variance. For the total population the probability of cosinor component (A*24 h) was significantly higher (63 ± 7 %) than that of the gaussian one (37 ± 7 %, p < 0.024) (Additional file 1 and Fig. 2). When total population was divided into excluding subgroups, proportion between probabilities (roughly 60 % vs. 40%) was maintained among most subgroups (p < 0.01 to p < 0.0001) except for older patients in which the difference between cosinor and gaussian was the highest (83 ± 7 % and 17 ± 7% respectively, p < 0.00001) (Additional file 1 and Fig. 3) and patients with previous symptoms whose cosinor and gaussian fitting resulted equally probable (Fig. 4).

The acrophase of the cosinor component occurred between 17:25 and 19:10 h in the total population and in the excluding subgroups while gaussian maximum was at 10:00 h with a SD of ± 2 h. Differences in these parameters between each pair of excluding subpopulations or against total population were non-significant.

## Discussion

Almost all cardiovascular events exhibit a pronounced circadian rhythm, with higher frequencies in the morning when patients wake up, resume upright posture and begin activities, and a relatively lower risk during sleep. These circadian rhythms, although anchored genetically, are synchronized and maintain certain phase relationships to external factors, especially the sleep portion of the light-dark schedule [28,29]. Such predictable-in-time differences in the physiological status of the cardiovascular system explain the rhythmic variations in the susceptibility of human beings to morbid and mortal events.

The foregoing results describing a cohort of 1063 patients recruited from 65 CCU located in Argentina and Uruguay who were admitted to the CCU within 24 h of the onset of symptoms of an acute MI, point out to the existence of two maxima in the incidence of MI, at the morning (mean value = 7.5 %/h between 08:00 and 12:00 h) and at the afternoon (mean value = 6 %/h between 16:00 and 20:00 h). A nadir in nocturnal MI incidence (1.3 %/h between 03:00 and 07:00 h) and a secondary minimum at early afternoon (3.8 %/h between 13:00 and 15:00 h) also occurred. The best fit curve (explaining ca. 85% of variance) was a 24 h cosinor (acrophase ~19:00 h, accounting for 63 % of variance) with a symmetrical gaussian "bell" (maximum at ~10:00 h, accounting for 37% of variance).

A similar bimodal picture was observed for MI frequencies among different excluding subgroups (older or younger than 70 years; with or without previous symptoms; diabetics or non diabetics; Q wave- or non-Q wave-type MI; anterior or inferior MI location). Proportion between cosinor and gaussian probabilities was maintained among most subgroups except for older patients who tended to have more MI at the afternoon and for patients with previous symptoms, who had MI equally distributed between the morning and the afternoon maxima.

Following the proposed model, the population of patients in the present examined sample could be divided into those having more risk of MI in the morning (described by the gaussian function), and in those having more risk during the evening (described by the 24 h cosinor function). For subjects in the "cosinor distribution", the amplitude of oscillation B resulted similar to the value of constant A (B/A ratio near unity), indicating a strong oscillation in MI episodes over the daily mean value of cosinor. Minimal (but not zero) MI incidence occurs at 07:00 h, which slowly rises up to maximum at 19:00 h and then slowly declines toward the minimum at 07:00 h.

For patients in the "gaussian distribution" a high incidence in MI was seen during the morning (maximum ~ 10:00 h). For these patients MI begins to occur at 04:00 h and attains its maximum at 10:00 h with a fast decline towards 16:00 h. In this group MI episodes resulted virtually absent between 16:00 and 04:00 h. At the evening, the observed MI's belong only to the "cosinor group" while during morning hours, most of recorded MI's were suffered by the "gaussian group" plus a minor fraction (slowly increasing as time elapsed) of the "cosinor group".

Time dependence and width of peaks (described by phase lag of the cosinor or time of mean and SD of gaussian adjust) remained the same in the different excluding subgroups extracted from total population. Likewise, the fraction of population associated to each component (approximately 40% gaussian/60% cosinor) was independent on the subgroup under consideration, except for patients older than 70 years and patients with previous symptoms. Indeed, older individuals appeared to double the chance to suffer a MI during the evening in comparison to morning hours (83% of the older patients suffered MI at evening vs. 17% in the morning). It should be noted that older and anterior infarct populations showed a significant increase in threshold above which the cosinor fitting is mounted. While the minimum for the different cosinors showed a mean value of 0.46 ± 0.44 %/h (SD) the two populations mentioned showed mean values that differed for more than 2 SD over the mean in a one-tailed normal test. This could be interpreted as the occurrence of extra risk factor (s) whose probability maintains constant during the day.

The high susceptibility of this Argentine and Uruguayan population to show MI at the evening points out to the relevance of variations in the spectrum of cardiovascular disease between different regions of the world and ethnic groups [21-25]. Indeed, it has been suggested that environmental-genetic background, socio-economic, and customs could underlie ethnic disparities in cardiovascular risk factor profiles [30,31]. A recent study confirms the existence of ethnic disparities in cardiovascular risk prevalence [25].

The prevalence of afternoon episodes in the present sample as compared to other studies could be related to a different daytime schedule, exposure to light, meals or disparities in cardiovascular risk factors, e.g. smoking. A prolonged siesta, which is a common practice in the present Argentine and Uruguayan population, could be followed by an increase in heart rate and blood pressure as it happens after waking up in the morning [32], leading to an increase in cardiovascular events. Afternoon siesta, in fact, may act as a triggering factor for cardiac events, especially in elderly subjects [33,34]

Changes in heart rate, blood pressure, neural and humoral vasoactive factors and heart contractility are presumably involved in the increase of myocardial oxygen demand that predisposes to the ischemic attack [35-39]. In a recent paper we described the relationship between unstable angor and circadian periodicity of heart rate variability (HRV) in a group of patients hospitalized in the CCU. As compared to moderate angor patients, amplitude of 24 h variation of total power decreased in severely affected patients and the circadian oscillation of vagal control on the heart became free running [40]. To what extent free running of circadian rhythms in cardiovascular function accounts for the bimodal distribution of MI reported herein deserves to be further examined.

## Conclusion

We are aware of the limitations of the present study. Determination of time of MI based on symptom onset could be subject to a bias, because it is possible that some people could have had symptoms for some hours and have called an ambulance later [41]. Taking this in consideration, the higher susceptibility of Argentine and Uruguayan patients to MI at the evening points out to the existence of significant variations in the spectrum of cardiovascular disease between different regions of the world and ethnic groups. Recognition of particular regional circadian patterns in myocardial ischemia is important in planning treatment strategies for patients with coronary artery disease to prevent the occurrence of sudden, catastrophic cardiac events.

## Competing interests

The author(s) declare that they have no competing interests.

## Authors' contributions

All authors have made substantial contributions to design of the analysis and the interpretation of data; and have been involved in drafting the article and revising it critically for important intellectual content; and have given final approval of the version to be published.

## Pre-publication history

The pre-publication history for this paper can be accessed here:



## Supplementary Material

Additional File 1 Curve fitting for MI probability density to MI incidence. Curve fitting for MI probability density to MI incidence for the total population of patients as for the excluding subgroups. Click here for file
