# Supplementary material for: Circadian analysis of myocardial infarction incidence in an Argentine and Uruguayan population
Source: BMC Cardiovasc Disord. 2006 Jan 9;6:1. doi: 10.1186/1471-2261-6-1 (PMC1360093; doi:10.1186/1471-2261-6-1)
Supplement: Additional File 1 — Curve fitting for MI probability density to MI incidence. Curve fitting for MI probability density to MI incidence for the total population of patients as for the excluding subgroups. [file 1471-2261-6-1-S1.doc]

**Table 2**: Curve fitting for MI probability density to MI incidence for the total population of patients as for the excluding subgroups.

| **SUBGROUPS** | **N** | **Cosinor**  **A + B * cos(ω_24_ * (t -t_acrophase_))** | | | | **Gaussian**  **(C/((2*Π)^1/2^* σ)) * exp-((t-t_m_)2/ 2* σ^2^)** | | | **P (cosinor)**  **vs P (gaussian)** | **Fit** | |
| --- | --- | --- | --- | --- | --- | --- | --- | --- | --- | --- | --- |
|  |  | **A**  **(%/h)** | **B**  **(%/h)** | **t_acrophase_**  **(hh:mm)** | **A*24 h (%)** | **C**  **(%)** | **t_m_**  **(hh:mm)** | **σ**  **(hh:mm)** | **(A * 24h. vs 50%)**  **(C vs 50%)** | **r** | **% of variance explained** |
| **Total population** | 1063 | 2.6±0.3 | 2.3±0.4 | 18:49±0:25 | 63±7 | 37±7 | 9:52±0:14 | 2:02±0:15 | p < 0.024 | 0.92 | 85 |
|  |  |  |  |  |  |  |  |  |  |  |  |
| **> 70 years old** | 213 | 3.5±0.3 | 2.2±0.5 | 17:24±1:02 | 83±7 | 17±7 | 10:05±0:31 | 1:37±0:26 | p < 0.00001 | 0.81 | 65 |
| **≤ 70 years old** | 850 | 2.4±0.2 | 2.4±0.4 | 19:08±0:41 | 58±4 | 42±4 | 9:50±0:16 | 2:05±0:14 | p < 0.023 | 0.89 | 80 |
|  |  |  |  |  |  |  |  |  |  |  |  |
| **No previous symptoms** | 639 | 2.8±0.3 | 2.4±0.5 | 18:50±0:38 | 66±6 | 34±6 | 9:47±0:20 | 2:01±0:17 | p < 0.005 | 0.89 | 79 |
| **With previous symptoms** | 424 | 2.3±0.3 | 2.3±0.5 | 18:55±0:48 | 56±7 | 44±7 | 9:59±0:20 | 2:06±0:17 | NS | 0.91 | 83 |
|  |  |  |  |  |  |  |  |  |  |  |  |
| **Diabetic** | 170 | 3.0±0.4 | 2.5±0.6 | 18:48±0:57 | 71±9 | 29±9 | 9:14±0:28 | 1:38±0:28 | p < 0.008 | 0.79 | 62 |
| **Non diabetic** | 893 | 2.7±0.3 | 2.2±0.4 | 18:43±0:43 | 64±6 | 37±6 | 10:02±0:20 | 2:01±0:16 | p < 0.016 | 0.91 | 83 |
|  |  |  |  |  |  |  |  |  |  |  |  |
| **Q type** | 910 | 2.7±0.37 | 2.3±0.4 | 18:38±0:40 | 64±6 | 36±6 | 9:54±0:16 | 2:02±0:16 | p < 0.015 | 0.91 | 83 |
| **Non-Q type** | 132 | 2.7±0.3 | 2.3±0.5 | 18:19±0:48 | 65±7 | 35±7 | 9:42±0:17 | 1:48±0:17 | p < 0.011 | 0.90 | 80 |
|  |  |  |  |  |  |  |  |  |  |  |  |
| **Anterior** | 520 | 3.0±0.2 | 1.8±0.4 | 18:24±1:00 | 72±6 | 28±6 | 10:06±0:16 | 1:40±0:16 | p < 0.0001 | 0.88 | 78 |
| **Inferior** | 480 | 2.5±0.2 | 2.5±0.3 | 18:18±0:40 | 61±4 | 39±4 | 9:44±0:18 | 2:08±0:16 | p < 0.006 | 0.91 | 83 |

Shown are means ± SEM of MI incidence (% / h) values, calculated as (100 * MI's per hour / total N). Cosinor and gaussian components are shown separately to illustrate the contribution of each parameter in fitting the function **y(t)**. Curve fitting = cosinor + Gaussian for n = 24 time points. The correlation coefficient r and the percent of total variance r^2^ explained are shown. P(cosinor) vs P(gaussian): comparison between total probability of cosinor component and total probability of gaussian component. assuming normal distribution. NS: non significant.
